# CORRELATES OF LIFE SATISFACTION AMONG MIDDLE-AGED AND OLDER BLACK ADULTS

**DOI:** 10.1093/geroni/igz038.2766

**Published:** 2019-11-08

**Authors:** Shyuan Ching Tan, Alyssa Gamaldo, Angie L Sardina, Ross Andel

**Affiliations:** 1 The Pennsylvania State University, University Park, Pennsylvania, United States; 2 The Pennsylvania State University, State College, Pennsylvania, United States; 3 University of North Carolina Wilmington, Wilmington, North Carolina, United States; 4 University of South Florida, Tampa, Florida, United States

## Abstract

This study explored satisfaction across life domains (e.g., family, daily life, health, finances, city of residence) and correlates of satisfaction across domains. Black adults (n=93, age range=55-80) completed the domains of life satisfaction scale and measures of sociodemographic factors, personality, and mental/physical health. Participants’ satisfaction was highest for home condition, but lowest for health. Univariate analyses of variance demonstrated better life satisfaction in the oldest-old (80+) than the youngest-old (55-64; p<.05), particularly in the domains of daily life/leisure, current financial situation, and total household income. Linear regression models suggested that higher satisfaction was associated with less education, less financial strain, lower depressive symptoms, and better self-rated physical health, although the pattern of results varied by domain. Satisfaction may increase with advancing old age, at least in some life domains. It can also vary across life domains and unique factors likely relate to satisfaction in each life domain.

